# Harnessing time: Leveraging chronotype asynchrony to promote efficient speech learning

**DOI:** 10.1121/10.0046951

**Published:** 2026-10-26

**Authors:** A. L. van Zelst, F. S. Earle

**Affiliations:** 1 Department of Communication Sciences and Disorders, Florida Atlantic University, Boca Raton, Florida 33431, USA; 2 Department of Communication Sciences and Disorders, University of Delaware, Newark, Delaware 19713, USA

## Abstract

This study examined chronotype synchrony effects on learning a nonnative speech contrast. Fifty-nine young adult native speakers of American English completed a circadian preference questionnaire and trained on producing a Danish vowel contrast in the morning or evening. Perceptual change (discrimination and identification, *n*** **=** **26) and production change (acoustic distance from a native model, *n*** **=** **59) were assessed. Identification improved with training, but improvement did not depend on circadian preference or training time. Production gains were greater when training occurred outside learners' preferred time, although the effect was small, suggesting that speech-motor learning may benefit from reduced executive control.

## 1. Introduction

Chronotype, or an individual's preferred timing of sleep and wakefulness, reflects circadian variation in physiological and cognitive functioning across the 24-h day (May *et al.*, 2023; Roenneberg *et al.*, 2003). Chronotype is distributed continuously, and young adults as a group are shifted toward eveningness (Adan *et al.*, 2012; Roenneberg *et al.*, 2004). Performance on tasks that rely heavily on executive control is generally enhanced when testing occurs in alignment with an individual's chronotype, a phenomenon known as the synchrony effect (May *et al.*, 2023; Schmidt *et al.*, 2007). In contrast, tasks that rely on implicit or procedural learning may benefit from non-optimal times of day, when reduced executive control is thought to lessen competition from explicit cognitive processes [Delpouve *et al.* (2014) and May *et al.* (2005); see also Smalle *et al.* (2021)]. The effects of time of day on speech learning may therefore depend on the cognitive processes supporting different aspects of learning.

Current models of speech-motor control propose that speech learning emerges through incremental adjustments to motor commands driven by discrepancies between predicted and realized sensory outcomes [e.g., Guenther (2006) and Houde and Nagarajan (2011)]. These adjustments depend on an accurate acoustic target, which guides the refinement of articulatory plans into increasingly stable patterns of speech production (Guenther, 2006; Houde and Nagarajan, 2011). Such feedback-driven adjustments can proceed outside of awareness (Munhall *et al.*, 2009) and are thought to depend on cortico-basal ganglia circuits that support procedural learning (Chang and Guenther, 2020). Consequently, successful speech learning requires both the establishment of an appropriate acoustic goal and the gradual optimization of the motor commands needed to achieve that goal.

Training paradigms involving nonnative speech sounds provide a useful framework for dissociating these complementary learning processes. Leading models of nonnative speech perception (e.g., Perceptual Assimilation model (Best and Tyler, 2007) and Speech Learning model (Flege *et al.*, 2021)] predict that nonnative speech sounds will tend to assimilate into neighboring, native speech categories. Establishing a novel phonetic category appears to draw on executive resources. Learners must redirect selective attention toward acoustic dimensions that are not weighted in their native language (Francis and Nusbaum, 2002), and dual-systems accounts propose that early speech category learning is dominated by a reflective system that depends on working memory and executive attention (Chandrasekaran *et al.*, 2014; Maddox and Chandrasekaran, 2014). In contrast, the gradual automatization of speech-motor movements may rely more heavily on implicit, feedback-driven learning mechanisms that are optimized when top-down executive control is reduced [see van Zelst and Earle (2021) for a theoretical review]. Because the availability of executive control varies with chronotype synchrony, the timing of training relative to circadian preference offers a noninvasive means of probing whether these two components of speech learning differ in their reliance on executive control.

The present study used chronotype synchrony to test whether perceptual and speech-motor learning during nonnative speech production training differ in their sensitivity to the time of day of training relative to a learner's circadian preference. Native English-speaking adults were trained to produce the Danish front rounded vowel contrast /y/-/ø/ in either the early morning or late evening, while changes in both perception and production were tracked across training. Circadian preference was measured with the Morningness–Eveningness Questionnaire [MEQ (Horne and Östberg, 1976)] and treated as a continuous predictor. We hypothesized that perceptual learning, reflecting the establishment of an accurate acoustic goal, would be enhanced when training occurred in synchrony with participants' circadian preference because of the greater availability of executive resources. In contrast, we predicted that speech-motor learning, reflected in the incremental refinement of articulatory movements toward the target acoustic goal, would be enhanced during non-optimal times of day, when reduced executive control may facilitate implicit sensorimotor learning.

## 2. Methods

The current manuscript features secondary analyses on a subset of data collected under the study described in van Zelst and Earle (2024), which concerned post-training, consolidation-mediated changes to production. The current paper focuses on perception and production during the training phase. Please refer to the prior paper for methodological details of the broader study.

### 2.1 Participants

The study was advertised for native speakers of English aged 18–25 years without a known history of cognitive, neurological, psychiatric, socio-emotional, attentional, or speech and/or language disorders, or use of psychotropic/psycho-attentional medication, who had access to a web-enabled device and headphones or earphones.

A total of 76 participants who met inclusionary criteria enrolled in the study. Of these, data from 10 participants were not included due to attrition, and data from two others were lost due to data corruption. Of these, the recordings of the production trials for 15 participants were not of sufficient quality for formant extraction and subsequent analyses. We report on the data from the remaining 59 participants (M_age_** **=** **21.19, SD = 1.73, 55 cisgender women and 4 cisgender men). All 59 were included in the production analyses. The perception analyses included the 26 of these participants who completed the post-training perception tasks immediately after training (see Sec. 2.3; 24 women, 2 men). The number of participants in each analysis, by time of day of training and chronotype category, is shown in Table 1.

**Table 1.** Number of participants in each analysis by time of day of training and chronotype category. Note that chronotype categories follow Horne and Östberg (1976): Evening = MEQ scores 16–41; Intermediate = 42–58; Morning = 59–86. Synchronous participants are Morning types trained at 8:00 a.m. and Evening types trained at 8:00 p.m.; Asynchronous participants are Morning types trained at 8:00 p.m. and Evening types trained at 8:00 a.m. The perception sample comprises the participants who completed the post-training perception tasks immediately after training and had complete MEQ data; all were also included in the production sample. MEQ = Morningness–Eveningness Questionnaire; TOD = time of day of training; AM = training at 8:00 a.m.; PM = training at 8:00 p.m.

Analysis	TOD	Evening	Intermediate	Morning	Total
Production	AM	8	24	3	35
	PM	7	14	3	24
Perception	AM	4	8	3	15
	PM	3	5	3	11

### 2.2 Materials

To obtain a measure of the participants' circadian preference, participants completed the Morningness-Eveningness Questionnaire [MEQ (Horne and Östberg, 1976)], a 19-item self-report measure yielding scores from 16 to 86, with higher scores indicating greater morningness. The MEQ has been validated to show high reliability with physiological measures of acrophase (Natale *et al.*, 2006).

The auditory stimuli were comprised of two, nonnative, high front rounded Danish vowels, **/**y**/** and **/**ø/. American English listeners tend to assimilate front rounded vowels to English back rounded vowels, **/**y**/** most often to **/**u**/** and mid front rounded vowels less consistently, often to **/**ʊ**/** (Levy and Strange, 2008; Strange *et al.*, 2004). Thus, although English listeners may hear a difference between these vowels, mapping each onto a distinct category and articulatory configuration is not supported by the native inventory, yet the vowels could be readily disambiguated within a single training session (Flege *et al.*, 2021). All stimuli were produced by a single native talker of Copenhagen Danish (an adult cisgender woman), video recorded in a quiet room in Denmark, who produced five repetitions of each vowel in isolation and in an [hVd] frame within the carrier phrase “jeg siger ______” (“I say ______”; Kartushina *et al.*, 2015). This closed set of five tokens per vowel was used for training and all assessments with the [hVd] tokens used only in the production task. Audio files were extracted from the videos, cut from the carrier phrase, and rescaled to match on mean amplitude (70 dB) and duration using praat (Boersma and Weenink, 2022, 2025). A still image of the talker's mouth during production of each vowel served as a nonorthographic category label, avoiding interference from English orthography [e.g., the letter “y” (Escudero *et al.*, 2008)], and as a visible cue to lip rounding (Hazan *et al.*, 2005). The images were not intended as a manipulation of speech-motor learning.

Stimulus presentation and response recording was controlled using Gorilla Experiment Builder^TM^ (Anwyl-Irvine *et al.*, 2020).

### 2.3 Procedures

Participants were recruited using physical and online recruitment flyers and social media posts, provided informed consent on the Gorilla platform under procedures approved by the University of Delaware Institutional Review Board, and were compensated in Amazon gift cards at $10/h.

Participants were randomly assigned to an 8 a.m. or 8 p.m. training. Upon enrollment, participants first completed a demographic information and a language history questionnaire to confirm study eligibility, then completed the Morningness-Eveningness Questionnaire. Participants were then asked to complete a microphone check (open sourced via the Gorilla^TM^ platform) prior to experimental tasks.

The experiment began with two tasks designed to measure baseline perception of the target Danish vowels. First, participants completed 10 trials of a discrimination task without feedback. After a 250 ms fixation cross, two vowels in isolation were presented with an inter-stimulus interval of 1 s, and participants clicked “same” or “different.”

Second, participants completed 10 trials of a perceptual identification task without feedback. Participants first listened to each auditory token while viewing the corresponding image of the talker's mouth during production. On each trial, a 250 ms fixation cross-was followed by a Danish vowel in isolation, and participants clicked the one of two mouth images that matched the vowel. No orthographic or phonetic (IPA) labels were displayed, and no audio accompanied the response images.

Participants were then asked to provide a baseline measure of production of the target vowels for 20 trials, 10 trials in isolation (5 of each target vowel) and 10 trials in an/hVd/frame. In each trial, participants were presented with an acoustic model of a native speaker of Danish. Participants were asked to imitate the model and instructed to record their production using a microphone icon on the screen.

During training, participants completed 320 total trials of production practice, organized in 16 blocks of 20 trials with one target vowel in isolation per block. On each trial, a 250 ms fixation cross-was followed by the auditory model of the target vowel with the image of the talker's mouth, and participants recorded their own production. No feedback on production accuracy was provided. After training, participants repeated the discrimination and identification tasks either immediately after training or 12 h after training, as part of the design of the broader study. To examine training-induced changes in perception without the additional influence of consolidation, we limited the perception analyses to the 26 participants who completed the post-training tasks immediately after training and had complete MEQ data.

### 2.4 Acoustic analysis of production files and calculation of acoustic distance

The first three formant values were extracted at the steady-state midpoint of each vowel (Kent and Vorperian, 2018; McAuliffe *et al.*, 2017; Pierrehumbert *et al.*, 2004) using praat (Boersma and Weenink, 2022, 2025) and two open-access scripts customized to standardize measurement. One that created TextGrids (Crosswhite, 2026) and one that measured F1, F2, and F3 (King, 2026).

To measure changes to production behavior during production training, F1-F2-F3 values were converted to Bark scale (Zwicker, 1961) using the Traunmüller formula (Traunmüller, 1990) in the hqmisc package (Quené, 2022) in r (R Core Team, 2021), and the Euclidean distance between the participant's production and the Danish model in this three-dimensional space was computed for every trial. We did not use the Mahalanobis distance, as participants were imitating a specific acoustic target rather than approximating a learned category distribution.

### 2.5 Chronotype synchrony

Participants' chronotypes (Morning, Intermediate, Evening) were characterized by responses on the Horne and Östberg Morningness-Eveningness questionnaire (Horne and Östberg, 1976) using the standard cutoffs (Evening: 16–41; Intermediate: 42–58; Morning: 59–86). This resulted in 6 Morning, 15 Evening, and 38 Intermediate-type participants in the production sample (Table 1). MEQ scores ranged from 28 to 71 (production sample: M = 47.59, SD = 9.04; perception sample: M = 49.00, SD = 11.70). We use “MEQ score” for the continuous measure entered in all models, and “chronotype” for the categorical groupings, which were used only descriptively.

There is not a widely accepted definition for the time window of chronobiological alignment, however the Evening vs Morning task design employed in the current paper aligns with prior works that have investigated synchrony effects on other cognitive functions (May *et al.*, 2023; Schmidt *et al.*, 2007). For descriptive purposes, Morning types trained at 8 a.m. and Evening types trained at 8 p.m. were categorized as “Synchronous,” those whose chronotype was opposite to their training time as “Asynchronous,” and Intermediate types as a separate group (see Sec. 4). Because these groups were small and unequal (Table 1), all inferential analyses treated MEQ score as a continuous variable in interaction with the time of day of training [(TOD) AM vs PM].

## 3. Analyses and results

Synchrony effects on outcome measures were tested through a mixed effects modeling approach (VanLeeuwen *et al.*, 1996) using the lme4 package (Bates *et al.*, 2015) in r (R Core Team, 2021). Marginal ( $R_{m}^{2}$) and conditional ( $R_{c}^{2}$) coefficients of determination were calculated using the performance package (Lüdecke *et al.*, 2021), and p values for linear mixed models were based on Satterthwaite's approximation [lmerTest (Kuznetsova *et al.*, 2017)]. Categorical variables were effects coded (TOD: AM** **=** **−0.5, PM** **=** **+0.5; Time: baseline** **=** **−0.5, post-training** **=** **+0.5; sex: female** **=** **−0.5, male** **=** **+0.5). MEQ score was mean-centered and scaled so that coefficients reflect a 10-point difference, and Trial was expressed in units of 100 trials (Trial 1 = 0); lower-order effects are therefore evaluated at the sample-mean MEQ score. The emmeans package (Lenth and Piaskowski, 2026) in r was used for *post hoc* testing of estimated marginal means and trends to examine significant interactions observed in the mixed models. Three-way interactions were decomposed within the omnibus models using emtrends, which estimates the slope of a continuous predictor at each level of TOD and directly tests whether these slopes differ. Separate subgroup models were not fitted.

### 3.1 Perceptual outcome

As discrimination performance was at or near ceiling at baseline (M = 86.9% correct; 16 of 26 participants at or above 90%), the predicted synchrony effect on perceptual learning was examined through performance on the identification task; a parallel model of discrimination accuracy revealed no significant effects (all *p*** **>** **0.28). The trial-by-trial accuracy on the identification task (520 trials from 26 participants) was modeled using a binomial generalized linear mixed effects model, with a three-way interaction between TOD, MEQ score, and Time (baseline vs post-training), and by-Token and by-Participant intercepts modeled as random effects. The resultant model (*AIC*** **=** **589.9, *BIC*** **=** **632.5, $R_{m}^{2}$** **=** **0.05, $R_{c}^{2}$** **=** **0.23) contained a significant effect of Time (*EST*** **=** **0.46, *SE*** **=** **0.22, *z*** **=** **2.11, *p*** **=** **0.035). Identification accuracy improved from baseline (predicted probability correct = 0.72, 95% CI [0.62, 0.81]) to post-training (0.80 [0.72, 0.87]). No other effects were significant (all *p*** **≥** **0.157), including the Time** **×** **TOD (*EST*** **=** **−0.58, *SE*** **=** **0.44, *z*** **=** **−1.32, *p*** **=** **0.187), Time** **×** **MEQ score (*EST*** **=** **0.24, *SE*** **=** **0.19, *z*** **=** **1.26, *p*** **=** **0.208), and Time** **×** **TOD** **×** **MEQ score interactions (*EST*** **=** **0.41, *SE*** **=** **0.38, *z*** **=** **1.09, *p*** **=** **0.275).

Consistent with the absence of a three-way interaction, emtrends indicated that the slope of MEQ score on training-induced change (post-training minus baseline, logit scale, per 10 MEQ points) did not differ reliably from zero for AM trainees (*EST*** **=** **0.03, *SE*** **=** **0.28, *z*** **=** **0.12, *p*** **=** **0.908) or PM trainees (*EST*** **=** **0.45, *SE*** **=** **0.26, *z*** **=** **1.70, *p*** **=** **0.088), nor between them (difference = 0.41, *SE*** **=** **0.38, *z*** **=** **1.09, *p*** **=** **0.275). Numerically, evening-oriented PM trainees were more accurate than morning-oriented PM trainees at baseline (MEQ slope** **=** **−0.49, *SE*** **=** **0.27, *z*** **=** **−1.81, *p*** **=** **0.071), leaving them less room to improve, but this pattern was not reliable. Thus, training improved identification accuracy, but we found no evidence that this improvement depended on MEQ score, training time, or their alignment (Fig. 1).

**Fig. 1.**
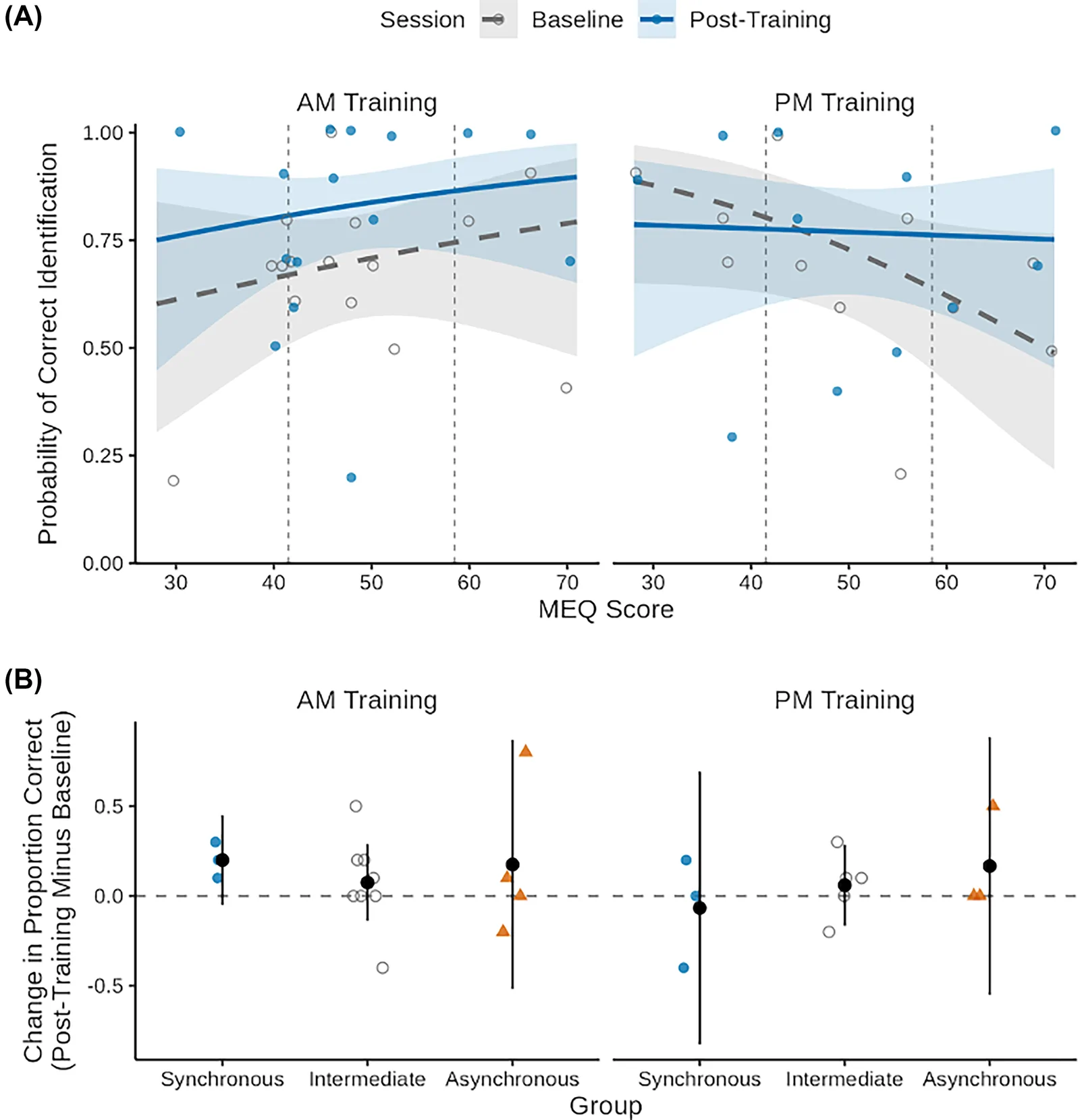
Identification accuracy by MEQ score, time of day of training, and session. Panel (A) shows the model-estimated probability of a correct identification at baseline (dashed line) and post-training (solid line) across the observed range of MEQ scores, from the generalized linear mixed effects model reported in the text. Shaded bands are 95% confidence intervals, and points are individual participants' observed proportions correct (10 trials per session). Dashed vertical lines mark the Horne and Östberg (1976) chronotype cutoffs (Evening ≤ 41; Morning ≥ 59). Panel (B) shows the observed change in proportion correct (post-training minus baseline) for Synchronous (Morning types trained at 8:00 a.m.; Evening types trained at 8:00 p.m.), Intermediate, and Asynchronous (Morning types trained at 8:00 p.m.; Evening types trained at 8:00 a.m.) participants; black points and error bars are group means and 95% confidence intervals. MEQ = higher scores indicate greater morningness; AM (n = 15); PM (n = 11).

### 3.2 Production outcome

To determine if chronotype synchrony affects learning rate for speech production, acoustic distance was modeled using a linear mixed effects model. The model contained a three-way interaction between TOD, MEQ score, and Trial number. Sex was modeled as a covariate, and by-Participant intercepts were modeled as a random effect. The resultant model (*AIC*** **=** **66640.2, *BIC*** **=** **66726.1, $R_{m}^{2}$** **=** **0.04, $R_{c}^{2}$** **=** **0.40) contained a significant effect of Trial (*EST*** **=** **−0.060 Bark per 100 trials, *SE*** **=** **0.012, *t*** **=** **−5.16, *p*** **<** **0.001), indicating that productions moved closer to the Danish model over the course of training. At the sample-mean MEQ score, the Trial** **×** **TOD interaction was not significant (*EST*** **=** **0.039, *SE*** **=** **0.023, *t*** **=** **1.67, *p*** **=** **0.096), but the three-way interaction between TOD, Trial, and MEQ score was (*EST*** **=** **−0.053, *SE*** **=** **0.026, *t*** **=** **−2.08, *p*** **=** **0.038), indicating that the relationship between MEQ score and the rate of change in acoustic distance differed between AM and PM trainees. No other effects were significant (all *p*** **≥** **0.087).

Decomposition with emtrends showed that among PM trainees, higher (i.e., more morning-oriented, and thus less synchronous) MEQ scores were associated with steeper decreases in acoustic distance across trials (*EST*** **=** **−0.035, *SE*** **=** **0.018, *t*** **=** **−1.98, *p*** **=** **0.048). At one SD above the mean MEQ score, distance decreased reliably (−0.072 Bark per 100 trials, *SE*** **=** **0.024, *z*** **=** **−3.01, *p*** **=** **0.003), whereas at one SD below the mean it did not (−0.009, *SE*** **=** **0.024, *z*** **=** **−0.39, *p*** **=** **0.697). Among AM trainees, the relationship was in the opposite direction but not significant (*EST*** **=** **0.018, *SE*** **=** **0.019, *t*** **=** **0.99, *p*** **=** **0.323). This crossover pattern, in which learners trained at a time misaligned with their circadian preference tended to show greater gains, is consistent with an asynchrony advantage for speech-motor learning (Fig. 2). The effect was small over 320 training trials. The model-estimated reduction in acoustic distance for PM trainees was 0.23 Bark at one SD above the mean MEQ score and 0.03 Bark at one SD below.

**Fig. 2.**
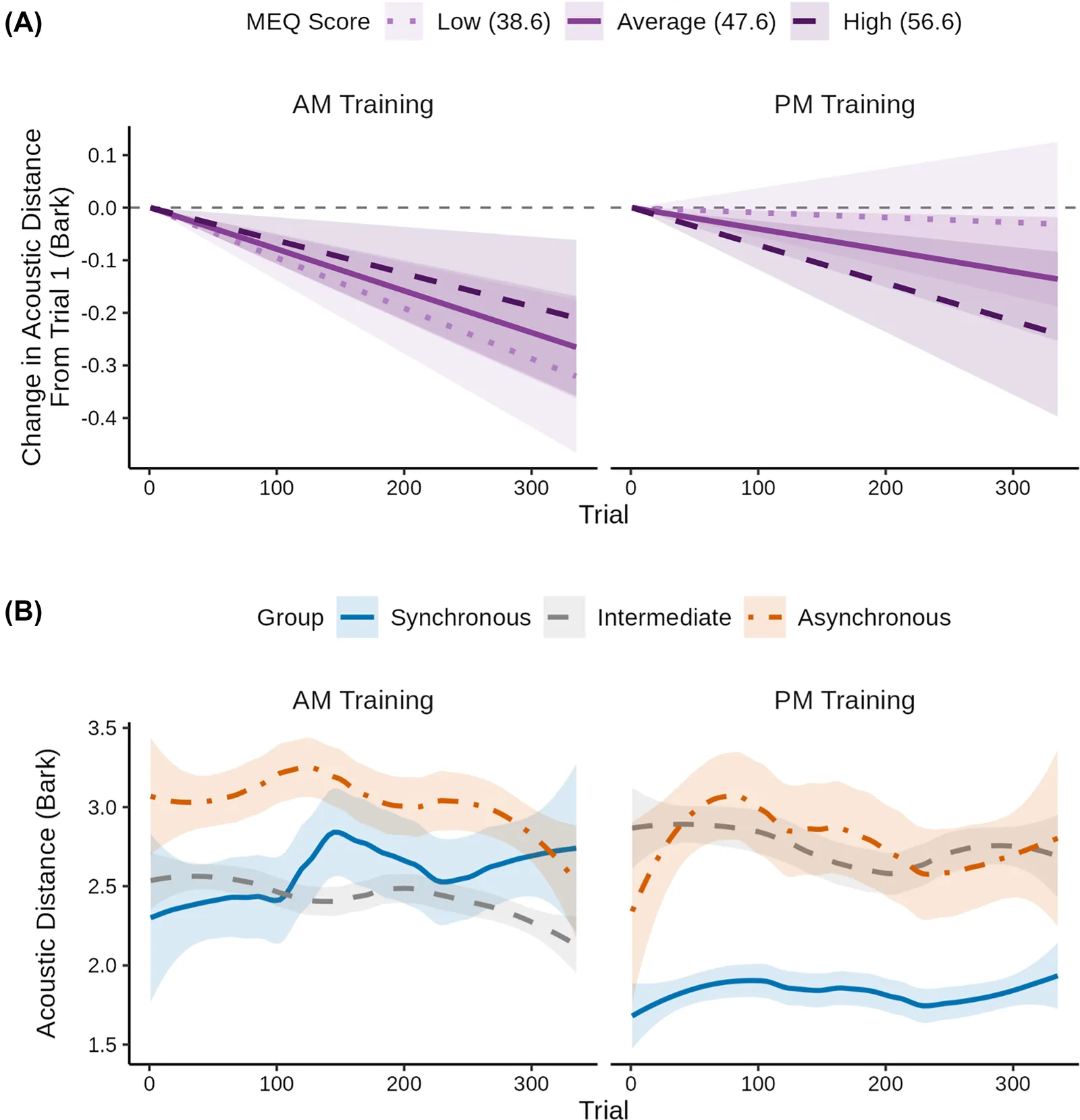
Change in acoustic distance across training trials by MEQ score and time of day of training. Panel (A) shows the model-estimated change in acoustic distance from Trial 1 at low (*M* − 1 *SD* = 38.6), average (*M* = 47.6), and high (*M* + 1 *SD* = 56.6) MEQ scores, from the linear mixed effects model reported in the text; negative values indicate productions closer to the native Danish model. Shaded regions are 95% confidence intervals of the estimated change. Panel (B) shows the observed acoustic distance across trials for Synchronous, Intermediate, and Asynchronous participants (defined as in Fig. 1), smoothed with locally weighted regression; shaded regions are 95% confidence intervals. MEQ = higher scores indicate greater morningness; AM (*n* = 35); PM (*n* = 24).

## 4. Discussion

Contrary to our first hypothesis, perceptual learning was not enhanced by chronotype synchrony. Identification improved with training, but not differentially by MEQ score, training time, or their interaction. Consistent with our second hypothesis, production learning depended on the alignment between training time and circadian preference. Among PM trainees, more morning-oriented (i.e., asynchronous) learners showed greater reductions in acoustic distance, with a nonsignificant opposite trend among AM trainees. Because MEQ score was modeled continuously, these results describe graded associations rather than comparisons of discrete groups, and the effects were small.

The null perception result should be interpreted with caution. It was based on 26 participants and 10 identification trials per session, discrimination was near ceiling, and all assessments used the training tokens. Evening-oriented PM trainees were also highly accurate at baseline, leaving little room to improve. Adequately powered studies with larger stimulus sets and generalization tests are needed.

The asynchrony advantage for production was reliable only among evening trainees. Among morning trainees, the trend was in the same direction but not significant. Larger samples of individuals with strong morning and evening chronotypes are needed to determine whether this asymmetry is reliable.

Most participants (38 of 59; 64%) were Intermediate types, consistent with the population distribution of chronotypes (Adan *et al.*, 2012). Intermediate types are not chronobiologically neutral. Among young adults, many lean toward eveningness (May *et al.*, 2023; Roenneberg *et al.*, 2004), and 8 a.m. may precede peak functioning even for some morning-oriented students (Evans *et al.*, 2017). “Asynchrony” for intermediate participants is thus a matter of degree, which the continuous MEQ analyses capture better than categorical groupings. Descriptively, Intermediate participants did not behave as a uniformly asynchronous group. Their perceptual gains and production trajectories overlapped with those of the other two groups [Figs. 1(B) and 2(B)], although with only 10 Synchronous and 11 Asynchronous participants in the production sample, these comparisons are exploratory. Future work should recruit more individuals with extreme chronotypes and schedule training relative to each participant's estimated circadian phase [e.g., mid-sleep time (Roenneberg *et al.*, 2003)] rather than at fixed clock times.

The asynchrony advantage observed for production is consistent with evidence that implicit learning benefits from non-optimal times of day and reduced executive control (Delpouve *et al.*, 2014; May *et al.*, 2005; Smalle *et al.*, 2021). It provides partial support for the dual-systems framework of speech-motor learning (van Zelst and Earle, 2021), which proposes distinct mechanisms for encoding an acoustic target and refining the motor program needed to achieve it. Refinement of the motor program may be optimized when attentional resources are not at their circadian peak, whereas whether target encoding benefits from executive control (Chandrasekaran *et al.*, 2014; Maddox and Chandrasekaran, 2014) could not be confirmed here. Thus, chronotype synchrony may not uniformly enhance learning, which is relevant to the timing of interventions that seek to maximize training-induced speech behaviors.

There were several limitations in this study. First, as a secondary analysis, our samples are not ideally balanced for sex or chronotype. Treating MEQ score as a continuous variable partly mitigates the latter, but the predominantly female sample may limit generalizability to men. Second, all stimuli came from a single talker, and the same five tokens per vowel, each vowel paired with a single still image, were used for training and all assessments, so participants may have learned talker-, token-, or image-specific associations, and generalization to novel tokens or talkers cannot be assessed. Third, formants were not normalized for vocal tract length, although by-participant intercepts likely absorbed some of this variability, and recordings made on participants' own devices may have added variability that led to underestimation of effects. Finally, acoustic distance is not the only measure of speech-motor learning, and other outcomes [e.g., identifiability of productions by native Danish listeners (van Zelst and Earle, 2024, 2026)] may not show the same effects.

## 5. Conclusion

Adults who are attempting to acquire a new speech-motor pattern may achieve better learning outcomes by practicing the target articulatory sequence at times of day that are misaligned with their circadian preference, although the effect observed here was small and requires replication. In contrast, we found no evidence that perceptual learning of the target contrast depended on the alignment between training time and circadian preference. These findings may point to a distinct role of reduced attentional control in speech-motor learning in adulthood, and may have scheduling implications for second language learning, as well as for clinical interventions for speech sound disorders.

## Data Availability

The critical data and code used in the statistical analyses are publicly available via the Open Science Framework (https://osf.io/yu2wq/overview?view_only=4662d0ff63794c8abfc6b8b5ed710945).
